# Counterfeits

**Published:** 1876-03

**Authors:** 


### COUNTERFEITS.

It requires more time, tact, and money to make a fraudulent enterprise remunerative than is requisite to amass a fortune by honest industry. Some men, however, are so intuitively mean, selfish, and grasping, that it seems impossible for them to do right. We have two or three such reprobates to deal with, and they are a constant source of annoyance, not only to us, but to many others whom they defraud out of small sums of money by launching at the beginning of each year "bogus" publications, called by some name calculated to deceive the public into believing that they are HALL'S JOURNAL OF HEALTH. This has been done in violation of our copyright, and in defiance of every principle of honor, honesty, and fair dealing. We are daily in receipt of letters from the victims of these frauds, who innocently supposed they were subscribing for HALL'S JOURNAL OF HEALTH. These fellows usually get out about two numbers in quick succession and then subside to enjoy their plunder, and elude our grasp upon them. They will find before the year closes that the latter will be a difficult thing to do. In sailor phrase, we have been giving them plenty of "lee way," and we promise that before the year ends they will have enough to do without getting out counterfeit JOURNALS OF HEALTH. We have cautioned our readers time and again to beware of these shams, and have not, therefore, a feeling of profound sorrow for the dupes who are daily writing to us for their promised "chromos," or the HALL'S JOURNAL OF HEALTH that was to be sent to them at 25 cents a year and upward.

It is hard to suppress a feeling of contempt for those who can be made to believe that the oldest and most popular Health Publication in the world begs subscriptions at 25 cents a year, or finds it necessary to resort to the "chromo dodge" to obtain subscribers.

It would be a sorry appreciation of all these years of labor which have bent our form and whitened our locks, were any of those whose names have stood on our books for a score of years to become suddenly clamorous for cheap chromos, in addition to the judicious advice we always strive to give them for the promotion of health and happiness.

In answer to at least a thousand persons who have asked us how to get the genuine HALL'S JOURNAL OF HEALTH, we reply, Direct all letters to the "EDITOR OF HALL'S JOURNAL OF HEALTH," No. 137 Eighth Street, New York. Do this whether the letters relate to the JOURNAL or to professional business. The editor attends to all the correspondence, and no other person is permitted to open the letters or to reply to them. All communications are strictly confidential, and will receive prompt attention.

---

SCRIBNER'S MONTHLY, for March, has an article on "Trinity College," Hartford, with fine illustrations, a biography of Honoré Belzac, and the first chapter of "The Mysterious Island" (condensed from Jules Verne).

